# Dynamic Transcriptome Analysis Reveals Potential Long Non-coding RNAs Governing Postnatal Pineal Development in Pig

**DOI:** 10.3389/fgene.2019.00409

**Published:** 2019-05-03

**Authors:** Yalan Yang, Rong Zhou, Wentong Li, Ying Liu, Yanmin Zhang, Hong Ao, Hua Li, Kui Li

**Affiliations:** ^1^Guangdong Provincial Key Laboratory of Animal Molecular Design and Precise Breeding, School of Life Sciences and Engineering, Foshan University, Foshan, China; ^2^Institute of Animal Sciences, Chinese Academy of Agricultural Sciences, Beijing, China

**Keywords:** pineal gland, pig, long noncoding RNA, postnatal development, transcriptome

## Abstract

Postnatal development and maturation of pineal gland is a highly dynamic period of tissue remodeling and phenotype maintenance, which is genetically controlled by programmed gene expression regulations. However, limited molecular characterization, particularly regarding long noncoding RNAs (lncRNA), is available for postnatal pineal at a whole transcriptome level. The present study first characterized the comprehensive pineal transcriptome profiles using strand-specific RNA-seq to illustrate the dynamic mRNA/lncRNA expression at three developmental stages (infancy, puberty, and adulthood). The results showed that 21,448 mRNAs and 8,166 novel lncRNAs were expressed in pig postnatal pineal gland. Among these genes, 3,573 mRNAs and 851 lncRNAs, including the 5-hydroxytryptamine receptors, exhibited significant dynamic regulation along maturation process, while the expression of homeobox genes didn’t show significant differences. Gene Ontology analysis revealed that the differentially expressed genes (DEGs) were significantly enriched in ion transport and synaptic transmission, highlighting the critical role of calcium signaling in postnatal pineal development. Additionally, co-expression analysis revealed the DEGs could be grouped into 12 clusters with distinct expression patterns. Many differential lncRNAs were functionally enriched in co-expressed clusters of genes related to ion transport, transcription regulation, DNA binding, and visual perception. Our study first provided an overview of postnatal pineal transcriptome dynamics in pig and demonstrated that dynamic lncRNA regulation of developmental transitions impact pineal physiology.

## Introduction

The mammalian pineal gland is a neuroendocrine transducer whose main and most conserved function is converting photoperiodic information into the nocturnal hormonal signal of melatonin synthesis and secretion (Maronde and Stehle, 2007). Melatonin regulates a variety of circadian and circannual physiological processes, such as the sleep-wake cycle, feeding, and cognition rhythms (Leon et al., 2004; Acuna-Castroviejo et al., 2007). Recent studies have revealed that melatonin also regulates many general physiological functions, including lipid and glucose metabolism, immune function, and carcinogenesis (Carrillo-Vico et al., 2005; Jha et al., 2015; Trivedi et al., 2016). Exploring pineal development will contribute to an improved understanding of its functions and mechanisms of regulation. The pineal gland develops as a tubular evagination from the dorsal diencephalon between the habenular and posterior commissures in the embryonic brain. The pineal gland displays a phase of rapid cell proliferation during the prenatal periods. However, cell proliferation activity terminates rapidly (Sapède and Cau, 2013), and pinealoblasts differentiate into pinealocytes during the two first postnatal weeks in rats (Calvo and Boya, 1983). After postnatal maturation, the parenchyma of the pineal gland is composed primarily of pinealocytes and interstitial cells (Moller and Baeres, 2002).

Pineal development is a complicated and dynamic process that is precisely genetically controlled by the programmed expression of gene cascades and TFs. Several TFs responsible for the establishment and maintenance of the pineal phenotype have been identified, such as the homeobox TFs *PAX6*, *LHX9*, and *OTX2* (Rath et al., 2013). However, gene abundance represents only part of the complexity of the transcriptome, as it has emerged that lncRNAs, which are a subgroup of transcripts that are longer than 200 nucleotides (nt) yet have limited protein-coding potential, have recently emerged as pivotal regulators in governing various developmental processes (Batista and Chang, 2013). For example, lncRNAs could regulate skeletal muscle differentiation during myogenesis, such as MyoD and H19 (Dey et al., 2014; Gong et al., 2015a). LncRNAs show precisely spatiotemporal expression patterns and regulate specific neuronal functions in brain (Briggs et al., 2015). Until now, the expression dynamics of mRNAs and lncRNAs involved in pineal gland have not been extensively explored. Describing the transcriptome profiles of the pineal gland through development may improve our understanding of the molecular pathways and regulatory mechanisms that are responsible for postnatal pineal development in mammals.

The pig (*Sus scrofa*) not only is an important agricultural animal but also serves as an attractive model organism for biomedical research, due to the similarity of its organ size, anatomy and physiology, and developmental processes with those of humans (Groenen et al., 2012; Prather, 2013; Niu et al., 2017; Yan et al., 2018). Hence, we can understand the developmental patterns of the pineal gland in mammals by using the pig as a model. In this study, we characterized high-resolution pineal transcriptome profiles in Y pigs using strand-specific total RNA sequencing, which allowed us to comprehensively illustrate the dynamic characteristics and functions of mRNAs/lncRNAs across three postnatal developmental stages: infancy (30 days, Y30), puberty (180 days, Y180), and adulthood (300 days, Y300). These results establish a general overview of the pineal transcriptome dynamics and pave the road for further investigations of the underlying functions and regulatory mechanisms of lncRNAs governing postnatal development of the mammalian pineal gland.

## Materials and Methods

### Sample Collection

Nine Y pigs with the same genetic background at postnatal days 30, 180, and 300 (three replicates per stage) were obtained from the Tianjin Ningheyuan Swine Breeding Farm (Tianjin, China) and slaughtered during daytime, between 10:00 and 14:00 Beijing time. The pineal sample of each pig was collected and immediately frozen in liquid nitrogen until RNA isolation. All animal procedures were performed according to the protocols of the Chinese Academy of Agricultural Sciences and the Institutional Animal Care and Use Committee.

### Transcriptome Library Preparation and Sequencing

Total RNA from pineal glands was isolated using TRIzol reagent (Invitrogen, Carlsbad, CA, United States) according to the manufacturer’s directions. The purified RNA was treated with DNase I (Qiagen, Beijing, China). The quantity and purity of the RNA samples were assessed using an Agilent 2100 Bioanalyzer (Agilent Technologies, CA, United States). Ribosomal RNA was depleted using the Epicentre Ribo-zero^TM^ rRNA Removal Kit (Epicentre, Madison, WI, United States). Next, strand-specific RNA-seq libraries for paired-end sequencing were prepared using the NEBNext^®^ Ultra^TM^ Directional RNA Library Prep Kit for Illumina^®^ (NEB, United States) according to the manufacturer’s instructions. Libraries were sequenced on an Illumina HiSeq 4000 platform to generate 150 bp paired-end reads (Novogene Bioinformatics Technology Co. Ltd., Tianjin, China).

### Transcriptome Assembly

The raw reads were firstly subjected to remove adaptor sequences and low-quality reads using custom scripts. The processed clean reads from each sample were then mapped to the reference genome of *Sus scrofa* (v11.1) using TopHat2 (v2.1.0) (Trapnell et al., 2009) with known gene annotation, parameters were set for strand-specific mapping (library-type “fr-secondstrand”). The reference genome sequence and gene annotation files were downloaded from the Ensembl database (release 90)^1^. After mapping, duplicate reads were removed using the rmdup tool in the samtools package (Li et al., 2009) to limit the influence of PCR artifacts. The remaining unique mapped reads of each sample were assembled into transcripts independently using Cufflinks (v1.3.0) (Trapnell et al., 2012) with the assistance of known annotations. Finally, assembled transcripts from each sample were merged into a consensus transcriptome using Cuffmerge (v1.0.0) (Trapnell et al., 2012).

### Identification of lncRNAs

**FIGURE 1:**
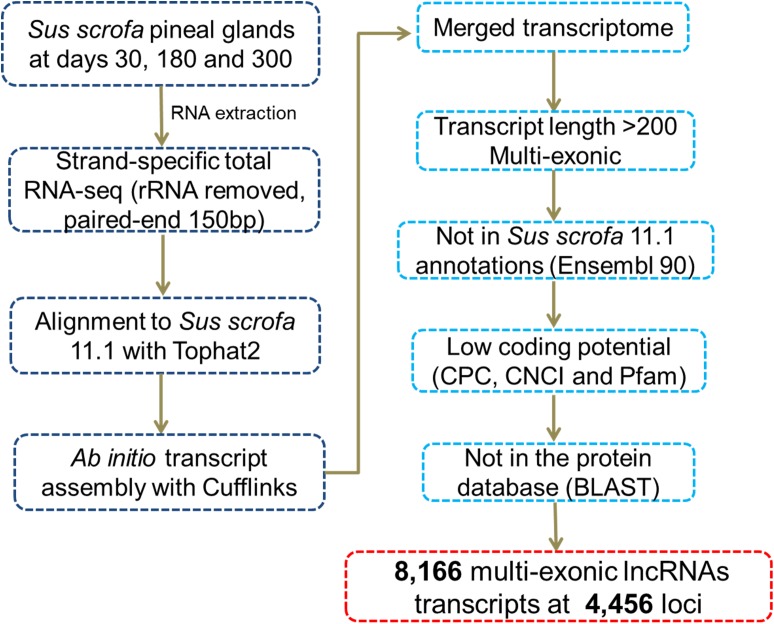
Pipeline for the identification of novel lncRNAs.

We identified novel lncRNAs in the pig pineal transcriptome using similar methods to those reported in our previous studies (Tang et al., 2017; Yang et al., 2017). A series of stringent filtering steps were utilized (Figure 1) as follows: (i) Single-exon transcripts and the transcripts less than 200 bp were removed to avoid unreliable transcripts; (ii) We filtered transcripts overlapping (>1 bp) with known gene models deposited in the Ensembl database; (iii) Coding Potential Calculator (CPC, v0.9-r2) (Kong et al., 2007) and Coding-Non-Coding Index (CNCI, v2) (Sun et al., 2013) programs were used to evaluate the coding potential of each transcript. Transcripts predicted to have coding potential (score > 0) by any of these two programs were filtered out; (iv) The transcripts whose corresponding translated protein sequences had a known protein-coding domain in the Pfam database (version 30.0) were removed by PfamScan (v1.3) (Finn et al., 2014); and (v) BLASTs (BLAST 2.2.26+) was used to remove transcripts with similarity to known proteins in the UniRef90 database (UniProt Consortium, 2015) with an *E*-value cutoff of 10^-5^. Transcripts remaining after the stringent filtering described above were considered putative lncRNAs.

### Expression Analysis

The raw read counts for each gene (mRNA/lncRNA) were calculated using HTSeq-count (Anders et al., 2015). For genes with multiple transcripts of different lengths, the longest transcript was selected to compute the gene expression level, measured as RPKM. Genes with RPKM ≥ 0.1 in at least one sample were defined as expressed genes. Highly expressed genes were defined as genes with a maximum RPKM ≥ 50 across the samples (Wang et al., 2014). The edgeR (exact test for negative binomial distribution) Bioconductor package (Robinson et al., 2009) in R software was used to identify DEGs between developmental stages. Gene expression normalization among samples to adjust for different sequencing depths across samples was performed using edgeR (Robinson et al., 2009). After estimating the dispersion of each gene, significantly DEGs were identified using cutoffs of false discovery rate (FDR) ≤ 0.05 and | log2 FC| ≥ 1 according to the edgeR’s recommendation (Robinson et al., 2009) and previous studies (Xue et al., 2013; Vanlandewijck et al., 2018).

### Co-expression Network Construction

Normalized, non-log transformed gene expression data (RPKM values) of all the differentially expressed mRNAs/lncRNAs were imported into Biolayout Express (3D) (Theocharidis et al., 2009). A pairwise gene-to-gene Pearson correlation matrix was calculated as a measure of similarity between genes. Based on a Pearson correlation coefficient cut-off threshold of *r* ≥ 0.90, a weighted, undirected co-expression network of mRNA-lncRNA interactions was generated. In this network, each node represents one gene (mRNA or lncRNA) and the edge between two nodes represents the Pearson correlation coefficients above the selected threshold. The network was clustered into groups of mRNAs/lncRNAs sharing similar expression patterns using the MCL algorithm (Enright et al., 2002), which has been demonstrated to be one of the most effective graph-based clustering algorithms available. To control the size of the clusters, the inflation coefficient was set to 2.4 and each cluster must contain at least 30 genes. This network was checked manually and clusters with no particular expression pattern were removed. Clusters were named according to their relative size, the largest cluster being designated Cluster 1.

### Functional Enrichment Analysis

Gene ontology enrichment analysis were performed by the DAVID website (v6.7^2^) (Huang et al., 2008) with a background set of human orthologues included in this study.

### Real-Time Quantitative PCR (RT-qPCR)

The total RNA of each sample was reverse transcribed into cDNA using a RevertAid First Strand cDNA Synthesis Kit (Thermo, Waltham, MA, United States) according to the manufacturer’s instructions. The RT-qPCR reaction solution was comprised of 10 μl of 2× SYBR Premix Ex Taq (Takara, Dalian, China), 0.4 μl of each primer, 1 μl of cDNA, 0.4 μl of Dye II, and sterile water to a volume 20 μl. The RT-qPCR cycling parameters were as follows: 95°C for 5 min, followed by 40 cycles at 95°C for 5 s and 60°C for 1 min. Next, a dissociation program was carried out at 95°C for 15 s, 60°C for 1 min, and 95°C for 15 s. Each reaction was performed in triplicate. The 2-ΔΔCt method was used to determine the gene expression level. The porcine *GAPDH* gene was selected as an internal control. All primer sequences are listed in Supplementary Table S1.

## Results

### Overview of the *Sus scrofa* Pineal Transcriptome Data

To identify changes in mRNA/lncRNA expression during postnatal pineal gland development, we generated RNA-seq libraries from the pineal glands of female Y pigs at infancy (Y30), puberty (Y180), and adulthood (Y300). Three biological replicates were evaluated per stage. Utilizing strand-specific RNA-seq of total RNA, a total of 1.05 billion clean sequencing reads (150 bp paired-end) were obtained after discarding low-quality and adaptor reads, corresponding to an average of 116.4 million sequence reads per sample. Of the clean reads, 79.9–90.0% could be mapped to the pig reference genome (version 11.1) by the Tophat2 pipeline (Trapnell et al., 2009) (Table 1). After removing duplicate reads, the remaining uniquely mapped reads were used for further lncRNA identification and gene expression analyses.

**Table 1:** Summary of sequencing metrics and read mapping for the RNA-seq of pig pineal glands.

Sample	Stage	Length	Reads	Mapped reads		Mapped ratio	
				
				Read1	Read2	Read1	Read2
Y30_1	30 days	150 bp	54,049,538 × 2	47,598,866	43,352,218	88.1%	80.2%
Y30_2	30 days	150 bp	50,796,645 × 2	44,735,289	41,004,165	88.1%	80.7%
Y30_3	30 days	150 bp	58,589,821 × 2	51,633,312	46,824,908	88.1%	79.9%
Y180_1	180 days	150 bp	79,812,929 × 2	70,250,218	67,703,427	88.0%	84.8%
Y180_2	180 days	150 bp	55,857,541 × 2	49,638,178	45,750,849	88.9%	81.9%
Y180_3	180 days	150 bp	58,459,709 × 2	51,560,101	46,995,972	88.2%	80.4%
Y300_1	300 days	150 bp	58,236,109 × 2	51,208,023	46,802,188	87.9%	80.4%
Y300_2	300 days	150 bp	53,832,028 × 2	48,429,903	44,651,018	90.0%	82.9%
Y300_3	300 days	150 bp	54,051,461 × 2	48,432,224	43,497,472	89.6%	80.5%


### Identification and Characterization of lncRNAs in the Pineal Transcriptome

After reconstructing the transcriptome using Cufflinks and Cuffmerge (Trapnell et al., 2009), we identified putative lncRNAs in the pineal transcriptome using a pipeline (Figure 1) similar to those reported in our previous studies (Tang et al., 2017; Yang et al., 2017). Eventually, a total of 8,166 multi-exonic lncRNA transcripts corresponding to 4,456 genomic loci were obtained (Supplementary Table S2). According to their genomic location, most (6,505) were lincRNAs located in intergenic regions, while 1,129 were lncRNAs transcribed from the antisense strand of the reference coding transcript, and the remaining 532 lncRNAs overlapped with middle coding exon regions.

**FIGURE 2:**
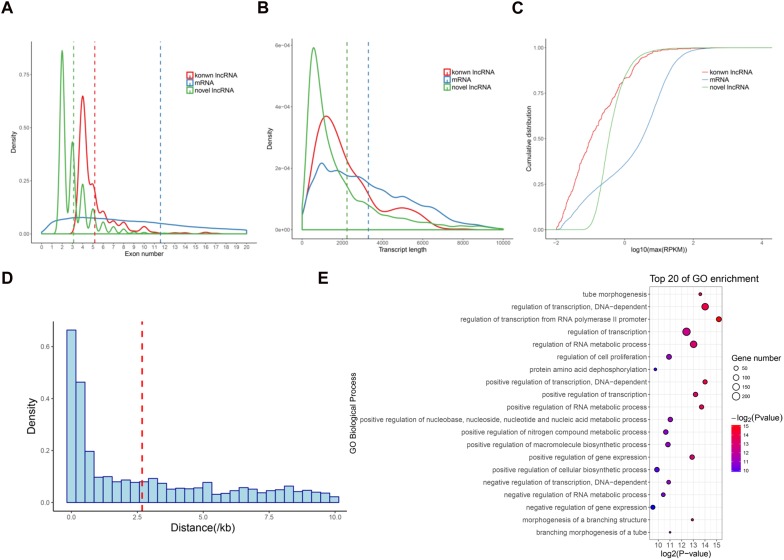
Characterization of lncRNAs in pig pineal glands. **(A)** Comparison of exon number between lncRNAs and mRNAs. **(B)** Comparison of transcript length between lncRNAs and mRNAs. **(C)** Comparison of expression level between lncRNAs and mRNAs. **(D)** The distribution of the distance from lincRNAs to their nearest neighboring protein-coding genes. The average (red dashed line) distance is indicated. **(E)** GO biological processes analysis of the neighboring protein-coding genes of the lincRNAs.

We next analyzed the features of these newly identified lncRNAs, namely novel lncRNAs. As expected, the novel lncRNAs contained fewer exons (3.1 exons on average) than mRNAs (11.6 exons on average; *P* < 2.2*e* − 16) (Figure 2A). The average transcript length of these lncRNAs (2235.2 nt) was significantly shorter than that of mRNAs (3296.1 nt; *P* < 2.2*e* − 16) (Figure 2B). Moreover, the expression levels of the lncRNAs (average RPKM = 2.7) were also significantly lower than those of the mRNAs (average RPKM = 10.9; *P* < 2.2*e* − 16) (Figure 2C). These results were consistent with the previous lncRNAs reports in pigs and other mammals (Iyer et al., 2015; Tang et al., 2017; Yang et al., 2017). Additionally, we found that 2,481 lincRNA were transcribed near (<10 kb) their protein-coding neighbors. The average distance from lincRNAs to their neighboring genes was 2.68 kb (Figure 2D). GO analysis revealed that these neighboring genes were significantly enriched in regulation of transcription and tube morphogenesis functions (Figure 2E), indicating that these lincRNAs are preferentially located in the vicinity of genes with specific functions that are closely associated with postnatal pineal development.

### Dynamic Expression of mRNAs and lncRNAs in Pineal Gland

**FIGURE 3:**
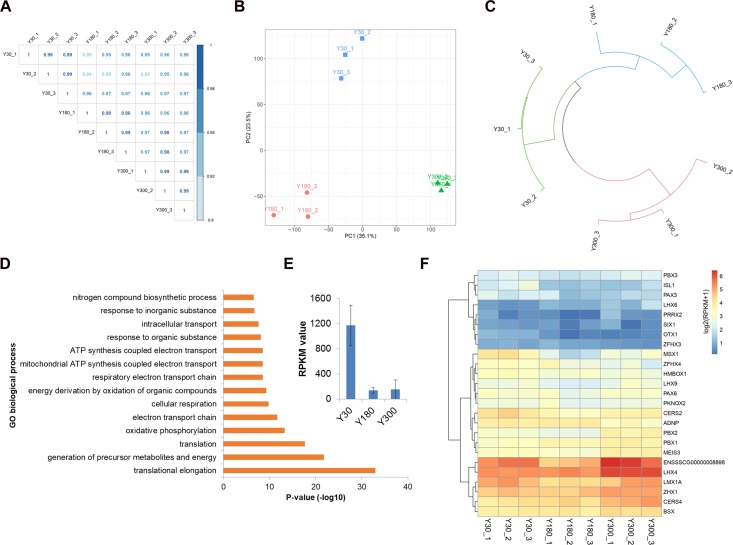
Dynamic expression profiles of mRNAs and lncRNAs during porcine postnatal pineal development. **(A)** Pearson correlation plot for the pineal transcriptome of Y30, Y180, and Y300, with three replicates for each developmental stage. **(B)** Principal component analysis of the pineal samples across three postnatal developmental stages based on both mRNA and lncRNA expression levels. Stages are illustrated by different shapes and colors. The *x*- and *y*-axes represent the first and second PC, respectively, with the percent variance explained by each PC in parentheses. **(C)** Hierarchical clustering analysis of the nine pineal samples across three developmental stages based on both mRNA and lncRNA expression levels. **(D)** Top enriched GO biological process terms of the highly expressed genes in the pineal gland. **(E)** The expression of *TTR* gene in postnatal pineal gland. **(F)** Heatmap showing the expression of homeobox transcription factors during postnatal pineal development.

We next evaluated the expression of novel lncRNAs, known lincRNAs, and mRNAs across postnatal pineal development and found a high Pearson correlation within and across stages (R > 0.95) (Figure 3A), indicating a high level of measurement consistency among biological replicates. A PCA was performed in order to understand the expression patterns of all mRNAs and lncRNAs during postnatal pineal development. We found that the PCA could clearly separate the three developmental stages from each other; the first two principal components (PC1 and PC2) could explain 35.1 and 23.5% of the transcriptional variation, respectively (Figure 3B). Clustering analysis revealed that samples within stages were clustered together first, and then Y30 and Y180 were grouped to form a larger cluster, and finally, clustered with Y300 (Figure 3C). These findings demonstrated a very high reproducibility within stages and distinct expression patterns across postnatal pineal development.

We detected an average of 15,388 mRNAs (a total of 21,448 mRNAs, with a range of 15,227–15,611 mRNAs per sample) and 2,740 lncRNAs (2,511–3,015 lncRNAs per sample) expressed (RPKM ≥ 0.1) in pineal glands, which accounted for 68.9 and 57.0% of the total mRNAs and lncRNAs, respectively. The RPKM values of most of the mRNAs were greater than 1, while the majority of the lncRNAs were lowly expressed (RPKM ≤ 0.1). Of these RNAs, 853 genes (842 mRNAs and 11 lncRNAs) were highly expressed in pineal glands (RPKM ≥ 50 in at least one sample). As expected, these genes were significantly enriched in translation, oxidative phosphorylation, and ATP synthesis-coupled electron transport functions (Figure 3D), all of which are essential for protein synthesis and other basic requirements for postnatal pineal development. Additionally, *TTR*, a pineal-specific gene, was highly expressed in our samples, especially at the Y30 stage (Figure 3E). Most of the homeobox TFs were lowly expressed in postnatal pineal gland (Figure 3F).

### Differentially Expressed mRNAs and lncRNAs

**FIGURE 4:**
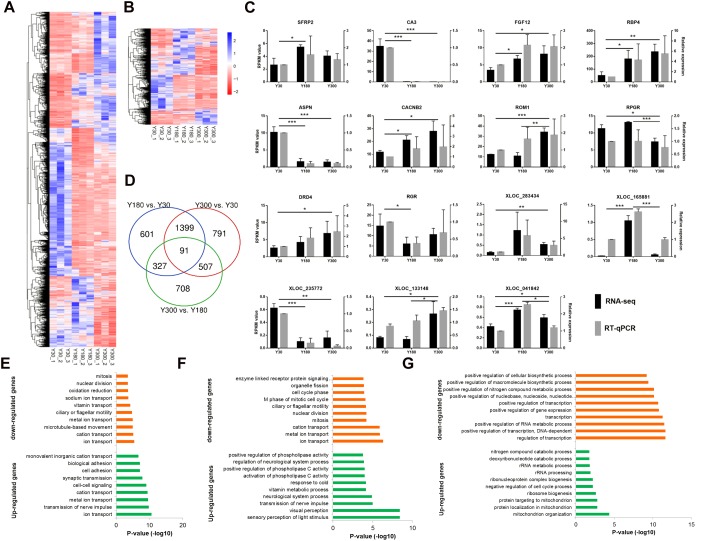
Differential expression analysis of mRNAs and lncRNAs during porcine postnatal pineal development. **(A,B)** Heatmap showing the differentially expressed mRNAs **(A)** and lncRNAs **(B)** during porcine postnatal pineal development. **(C)** Experimental validation of RNA-seq data by RT-qPCR. Gene expression differences between developmental stages were evaluated based on the RNA-Seq data using the edgeR package. Error bars are SEM, *n* = 3. ^∗^*P* < 0.05, ^∗∗^*P* < 0.01, ^∗∗∗^*P* < 0.001. **(D)** Venn diagram showing the number of differentially expressed mRNAs and lncRNAs between different development stages. **(E–G)** GO biological process analysis of the up-regulated and down-regulated genes between Y180-Y30 **(E)**, Y300-Y30 **(F)**, and Y300-Y180 **(G)**.

We found a total of 4,424 genes (including 3,573 mRNAs and 851 lncRNAs) with a significant difference in expression (|log_2_ fold change (FC)|≥1 and FDR≤0.05) between developmental stages, including 2,417 Y180-Y30 (including 1,982 mRNAs and 436 lncRNAs), 2,788 Y300-Y30 (including 2,264 mRNAs and 524 lncRNAs), and 1,633 Y300-Y180 (including 1,187 mRNAs and 446 lncRNAs) DEGs (Figure 4A,B). Several 5-hydroxytryptamine (serotonin) receptors were included in this list, including *HTR2A*, *HTR2B*, *HTR2C*, and *HTR7*. We randomly verified 15 of the DEGs (10 mRNAs and 5 lncRNAs) by RT-qPCR and found a high concordance between the RT-qPCR and the RNA-seq data (Figure 4C), suggesting that the differential expression analysis based on the RNA-seq data was reliable. The highest number of DEGs was observed in the Y300-Y30 comparison, which was correlated with the difference in development time among the three stages. Most DEGs were observed in at least two of the three comparisons, and 91 of them (56 mRNAs and 35 lncRNAs) were found in all three comparisons (Figure 4D), including genes related to phosphate metabolic (*PPM1J*, *ND4*, *PRLR*, and *ND5*) and cell motility (*CCK*, *FOXJ1*, *DCDC2*, and *DNAH2*). We further examined the enriched functions of the DEGs through GO enrichment analysis. Compared with Y30, the up-regulated genes in Y180 were significantly enriched in ion transport, transmission of nerve impulse, cell-cell signaling, and synaptic transmission functions, while the down-regulated genes were associated with ion transport, oxidation-reduction, and cell cycle categories (Figure 4E and Supplementary Table S3). The up-regulated genes in Y300 when compared with Y30 were significantly enriched in sensory perception of light stimulus, visual perception, transmission of nerve impulse, and neurological system process functions, while the down-regulated genes were enriched in ion transport and cell cycle functions (Figure 4F and Supplementary Table S3). Compared with Y180, the up-regulated genes in Y300 were significantly enriched in mitochondrion organization, ribosome biogenesis, and negative regulation of cell cycle process functions, while the down-regulated genes were associated with transcription and RNA metabolic and phosphate metabolic processes (Figure 4G and Supplementary Table S3).

### Inference of Pineal lncRNA Function Using Co-expressed Network

**FIGURE 5:**
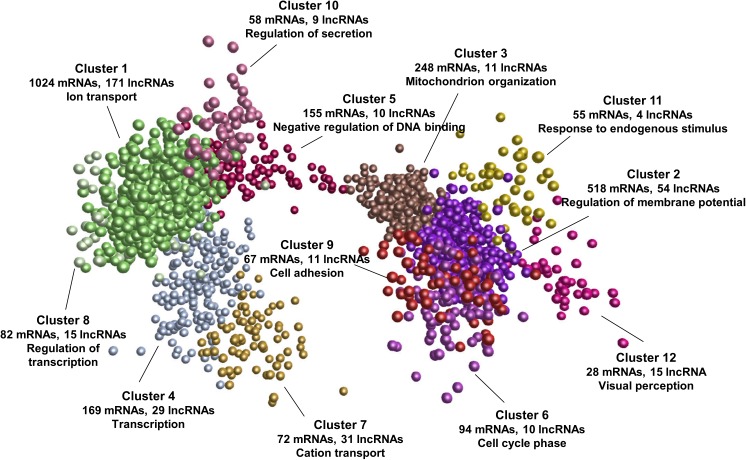
mRNA-lncRNA co-expression network. All the differentially expressed mRNAs and lncRNAs were used to construct the co-expression network by Biolayout Express (3D). The number of mRNAs/lncRNAs in each co-expression cluster and the most significantly enriched GO biological process of each cluster were shown.

To explore the potential functions and regulatory mechanisms of lncRNAs during postnatal pineal development, we constructed a co-expression interaction network of differentially expressed mRNAs and lncRNAs. The network consisted of 605,831 interaction pairs. These genes were grouped into 12 co-expression clusters by MCL algorithm (Enright et al., 2002). The expression pattern of each cluster during postnatal pineal development was shown in Supplementary Figure S1. Some of these clusters contained mRNAs that are closely associated with postnatal pineal development (Figure 5 and Supplementary Table S4). Cluster 1 was the biggest one, which contained 1024 mRNAs and 171 lncRNAs, genes in this cluster were highly expressed at Y30 stage, such as members of the solute carrier family genes (*SLC5A5*, *SLC13A5*, and *SLC39A12*). Cluster 7 was highly expressed at Y180 stage and contained 72 mRNAs and 31 lncRNAs. Interestingly, GO enrichment analyses suggested that ion transport was the most significantly enriched term of genes in these two clusters. The genes in both cluster 2 (518 mRNAs and 54 lncRNAs) and cluster 3 (248 mRNAs and 11 lncRNAs) were abundantly expressed at Y300 stage. The genes in cluster 3 were higher expressed at Y30 than at Y180 stage, while the genes in cluster 2 were stably expressed at these two stages. Cluster 2 and cluster 3 mainly functioned in regulation of membrane potential and mitochondrion organization, respectively. Additionally, cluster 4 (169 mRNAs and 29 lncRNAs) was enriched with transcription and oxidative phosphorylation genes, including the core subunits of mitochondrial membrane respiratory chain NADH dehydrogenase (*ND1*, *ND2*, *ND4*, and *ND5*). The genes in cluster 4 were higher expressed at Y180 stage than at Y30 and Y300 stages. Whereas the genes in cluster 5 exhibited an inversed expression patterns with the genes in cluster 4. Negative regulation of DNA binding was the most enriched biological process for cluster 5, which contained 155 mRNAs and 10 lncRNAs, such as *PTHLH*, *SMO*, *ID1*, and *XLOC_050558*. Genes in cluster 6 (94 mRNAs and 10 lncRNAs) were specifically expressed at Y300 stage, which were closely associated with cell cycle phase and mitosis, such as *CCNB1*, *CDC20*, and *DLGAP5*. Remarkably, the expressions of genes in cluster 9 (67 mRNAs and 11 lncRNAs) and cluster 10 (58 mRNAs and 9 lncRNAs) were continuously increased during postnatal pineal development, cell adhesion and regulation of secretion was the most significantly enriched biological processes in these two clusters, respectively. The continuously decreased genes were grouped into cluster 8 (82 mRNAs, 15 lncRNAs) and mostly enriched in regulation of transcription, such as *PLAG1*, *ATF7IP*, and *CRTC3*. Genes in cluster 11 and cluster 12 were associated with response to endogenous stimulus and visual perception, respectively. These results suggested putative regulatory functions for a subset of lncRNAs in postnatal pineal development.

## Discussion

In this study, we provided deep strand-specific RNA-seq of total RNA from three representative postnatal developing stages (infancy, puberty, and adulthood) of the porcine pineal gland, which we used to study the expression profiles of mRNAs/lncRNAs. We expect that this new resource will contribute to the understanding of the importance of transcription regulation in mammalian postnatal pineal gland development and maturation.

Pineal gland is an neuroendocrine organ for the regulation of the circadian clock system in all vertebrate species (Macchi and Bruce, 2004). It’s well known that homeobox genes are essential for normal pineal development and are key regulators in the maintenance of the postnatal pineal phenotype (Rath et al., 2013). We observed that most of the homeobox genes were lowly expressed (RPKM < 1) in our study, *HOPX* and *LHX4* were the most abundantly expressed ones in pig postnatal pineal gland, implying these two genes might play important roles in pineal development, though their function in pineal has not been reported. *LHX9* and *PAX6* are essential for early development of the mammalian pineal gland (Rath et al., 2013; Yamazaki et al., 2015), our study confirmed that these two genes were lowly expressed in the postnatal pineal gland after 30 days. *OTX2* displayed decreased expression in the postnatal pineal gland of rat (Rath et al., 2006), and was barely expressed in our samples. Additionally, the neurogenic differentiation factor 1 (*NEUROD1*) gene, a member of the bHLH TF family, is known to influence the fate of specific neuronal and endocrine cells (Munoz et al., 2007), and we confirmed that it was highly expressed in the postnatal pineal gland.

The expression of most DEGs changed constantly across postnatal pineal development, reflecting a dynamic regulation of gene expression. For example, the expression level of the transthyretin (*TTR*) gene, the major thyroid hormone transporter in the CNS, was much higher at Y30 than at Y180 or Y300. *TTR* has also been reported to be differentially expressed between midnight and midday in the pineal gland (Acuna-Castroviejo et al., 2007). We observed that DEGs were significantly associated with the ion transport, cell-cell signaling, synaptic transmission, and developmental maturation. Especially, 48 genes in calcium signaling pathway were differentially expressed, such as *CALB1* and *CACNB2*, which are involved in a variety of calcium-dependent processes, including cell motility, cell division, and hormone or neurotransmitter release (Dolphin, 2007). It’s reported that melatonin could modulate neural development through the regulation of calcium signaling (Poloni et al., 2011). Additionally, 88 DEGs were involved in transmission of nerve impulse, such as *HTR2A*, *HTR2C*, and *HTR7*, which are 5-hydroxytryptamine (serotonin) receptors. 5-hydroxytryptamine is a precursor for melatonin production and is produced abundantly in the pineal gland of all vertebrate animals (Sapède and Cau, 2013). These results provide evidences that the critical roles of ion transport, especially calcium signaling, in postnatal pineal development, which might contribute to deeply understand the complexity of the pineal architectures and functions.

With the rapid adoption of RNA-seq technologies, thousands of lncRNA in the genome have been discovered in various species, their functions in various biological processes have been demonstrated (Geng et al., 2013; Gong et al., 2015b; Volders et al., 2015; Liang et al., 2018). However, compared with those of human and mouse, the lncRNA resources in pig are relatively limited (Quek et al., 2015; Liang et al., 2018). In this study, we identified a total of 8,166 novel lncRNAs, greatly expanding the genomic information of non-coding RNAs in pigs. These lncRNAs exhibited similar genomic characteristics with those of lncRNAs described in previous studies of pigs and other mammals (Iyer et al., 2015; Tang et al., 2017; Yang et al., 2017). 851 lncRNAs, including 35 known and 816 novel lncRNAs, were differentially expressed across postnatal pineal development. Remarkably, 282 of them were transcribed near their protein-coding neighbors. For instance, XLOC_199747 located upstream of neurotrophin 3 (*NTF3*). There was a significantly positive correlation between the expressions of these two genes (*r* = 0.81). These results suggested that these differentially expressed lncRNAs might act on mRNAs involving in postnatal development by *cis* regulation. Co-expression analysis identified coordinated gene clusters that were shared in a developmental-specific expression fashion, which is an effective approach to uncover the function of lncRNAs (Pauli et al., 2012; Anamaria et al., 2014). We found most clusters containing genes with interesting functions. For example, GO enrichment analyses suggested that the cluster 1 was mostly associated with ion-transport, including many solute carrier family genes, which play important roles in the adrenergic regulation of cAMP and cGMP in pinealocytes (Sugden et al., 1986). Genes in cluster 2 were highly expressed at Y300 stages, which were closely related to regulation of membrane potential and transmission of nerve impulse, implying the critical roles of these mRNAs (such as *SCN1B* and *SYT4*) and lncRNAs (such as *XLOC_018250* and *XLOC_179558*) in mature pineal gland. Cell cycle genes (such as *CCNB1*, *CCNB2*, *CCNB3*, and *CCNF*), and 10 lncRNAs (such as *XLOC_280714* and *XLOC_156756*) were grouped into Cluster 6. The expression of these genes was decreased dramatically during postnatal pineal development, which was in consistence with the termination of pinealoblasts proliferation after birth (Sapède and Cau, 2013). Another intriguing example is cluster 12, which includes 15 lncRNAs (such as *XLOC_046348* and *XLOC_196944*). The mRNAs in this cluster were enriched in functional terms related to visual and sensory perception, which have been shown to play essential roles in circadian melatonin rhythm (Reiter, 1993). The dynamic changes observed in the co-expression networks offer insights regarding to the functions and regulation of lncRNAs during postnatal pineal development.

## Conclusion

Overall, our data cataloged the pineal transcriptional profiles and basic gene expression features during postnatal development and maturation in pig. Novel lncRNAs were identified, which provide rich resources for understanding the molecular mechanisms and regulatory network of postnatal pineal development in mammals. The lncRNAs in the co-expression network may be considered as promising targets for postnatal pineal development, maturation, and phenotype maintenance, but their function still needs to be further explored at the molecular, cellular, and individual levels.

## Ethics Statement

All animal procedures were performed according to the protocols of the Chinese Academy of Agricultural Sciences and the Institutional Animal Care and Use Committee.

## Author Contributions

KL designed and managed the project. YY administered the computational analysis. YY and RZ analyzed the data and wrote the manuscript. RZ, WL, YL, YZ, and YY performed animal work and collected biological samples. WL, YL, and YZ performed molecular experiments. HL, HA, and KL revised the manuscript. All the authors approved the final manuscript.

## Conflict of Interest Statement

The authors declare that the research was conducted in the absence of any commercial or financial relationships that could be construed as a potential conflict of interest.

## Footnotes

1 http://asia.ensembl.org/index.html

2 http://david.abcc.ncifcrf.gov/

## Supplementary Material

Figure S1 The dynamic expression pattern of each co-expression cluster during postnatal pineal development.

Table S1 Primer sequences of mRNAs and lncRNAs selected for validation by RT-qPCR.

Table S2 Summary of the predicted lncRNAs in the *Sus scrofa* pineal gland.

Table S3 GO biological process enrichment analysis of the differentially expressed genes between different developmental stages.

Table S4 GO biological process enrichment analysis of the mRNAs in each co-expression cluster.

## Abbreviations

DAVID: database for annotation, visualization, and integrated discovery
DGE: differentially expressed gene
GO: gene ontology
lincRNA: long intergenic noncoding RNAs
lncRNA: long noncoding RNAs
MCL: Markov clustering
PCA: principal component analysis
RPKM: reads per kilobase per million reads
RT-qPCR: real-time quantitative PCR
TF: transcription factor
Y: Yorkshire
