# Supplementary figures and images for: Dynamic Transcriptome Analysis Reveals Potential Long Non-coding RNAs Governing Postnatal Pineal Development in Pig
Source: Front Genet. 2019 May 3;10:409. doi: 10.3389/fgene.2019.00409 (PMC6510172; doi:10.3389/fgene.2019.00409)

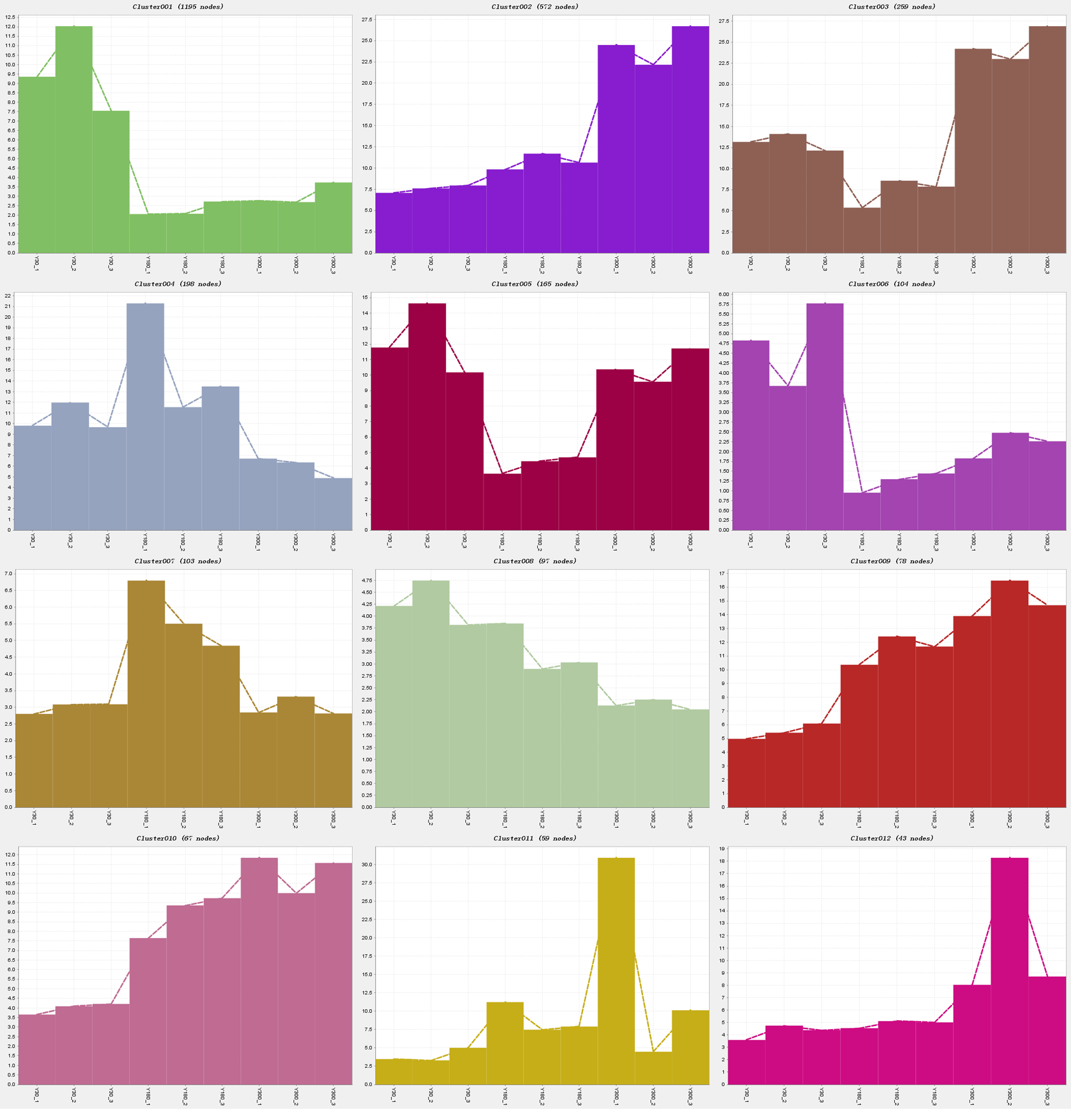

Supplement: Figure S1 — The dynamic expression pattern of each co-expression cluster during postnatal pineal development. [file Image_1.TIF]
