# Supplementary material for: Dynamic Transcriptome Analysis Reveals Potential Long Non-coding RNAs Governing Postnatal Pineal Development in Pig
Source: Front Genet. 2019 May 3;10:409. doi: 10.3389/fgene.2019.00409 (PMC6510172; doi:10.3389/fgene.2019.00409)
Supplement: Table S1 — Primer sequences of mRNAs and lncRNAs selected for validation by RT-qPCR. [file Table_1.DOCX]

Supplementary Table 1. Primer sequences of mRNAs and lncRNAs selected for validation by qPCR.

| **Gene** | **Primer sequence (5’->3’)** |
| --- | --- |
| *SFRP2* | Forward: CGATGACCTGGACGAAACCA |
|  | Reverse: ATGCAGAGGTCGTTGTCCTG |
| *ASPN* | Forward: GCCAAACCCTTATTTCGCCC |
|  | Reverse: GTTGCTTGGGACAGAGGACA |
| *CA3* | Forward: CCAATCGCCCATTGAACTGC |
|  | Reverse: CCTGCAGGTCTTCCCATTGT |
| *CACNB2* | Forward: AACGTCACCCCACTCCAAAG |
|  | Reverse: TCGGTGACCTCATACCCCTT |
| *FGF12* | Forward: GATAGCCAGCTCCTTGATCCG |
|  | Reverse: GCACTTTGCTGAATACCCCG |
| *RBP4* | Forward: GACCCCGAGGGACTCTTTCT |
|  | Reverse: AAAGACGGACTCGACCCTTG |
| *ROM1* | Forward: CAAACGGCTGTTGGATGAGC |
|  | Reverse: TTGCTCTGGATCCGGTCAAC |
| *RPGR* | Forward: GACGTGGATGAGGAAACGGA |
|  | Reverse: TGGCTTTTTGGCCCTTTTCA |
| *DRD4* | Forward: ACGCCCACCAACTACTTCAT |
|  | Reverse: GTGCACCTCGGAATAGACGA |
| *RGR* | Forward: GCCCCTCTTCATCACAGTCAC |
|  | Reverse: CCCCAGGCGAGCATCAGT |
| *XLOC_283434* | Forward: CGACTTAGCCTGGCACCC |
|  | Reverse: GCTCCATCTTTGAACAGCACTC |
| *XLOC_165881* | Forward: GAAAGCCGTGAGGGAAAACA |
|  | Reverse: GTGGAAGCCAGTTGCCATG |
| *XLOC_235772* | Forward: TGGCAGCTCAGCTCCGATTA  Reverse: CACAAGGGAACTCCACTGGTTA |
| *XLOC_133148* | Forward: ATGAGGTTGCGGGTTTGATC  Reverse: CCAGGCTAGGGGTCTATTCG |
| *XLOC_041842* | Forward: GGAGCTGTCTTGCTTCAGTTTG  Reverse: GAATTAAACCAGGCAGGCAGT |
| *GAPDH* | Forward: AGGGCATCCTGGGCTACACT |
|  | Reverse: TCCACCACCCTGTTGCTGTA |
